# Prevalence of Hypertensive Disorders of Pregnancy and Associated Birth Outcomes Among Adolescent Mothers at a Tertiary Hospital in Zambia: A Retrospective Cross‐Sectional Study

**DOI:** 10.1155/ijhy/9947103

**Published:** 2026-07-02

**Authors:** Martin Chakulya, David Chisompola, Hanzooma Hatwiko, Lukundo Siame, Joreen P. Povia, Natasha Chishala, Annet Kirabo, Sepiso K. Masenga

**Affiliations:** ^1^ Department of Physiological Sciences, Mulungushi University School of Medicine and Health Sciences, Livingstone, Zambia; ^2^ Department of Cardiovascular Science and Metabolic Diseases, Livingstone Center for Prevention and Translational Science, Livingstone, Zambia; ^3^ Department of Molecular Physiology and Biophysics, Vanderbilt University, Nashville, Tennessee, 37232, USA, vanderbilt.edu; ^4^ Department of Medicine, Vanderbilt University Medical Center, Nashville, Tennessee, USA, vanderbilt.edu

**Keywords:** adolescent pregnancy, birth outcomes, gestational hypertension, hypertensive disorders of pregnancy, preeclampsia, Zambia

## Abstract

**Background:**

Hypertensive disorders of pregnancy (HDP) are a leading cause of maternal and perinatal morbidity, yet data on their burden among adolescent mothers in Sub‐Saharan Africa are limited. This study aimed to determine the prevalence of HDP and their association with birth outcomes among adolescent mothers at Livingstone University Teaching Hospital (LUTH) in Zambia.

**Methods:**

We conducted a retrospective cross‐sectional study of 405 medical records of adolescent pregnancies (aged 10–19 years) at LUTH from January 2023 to December 2024. HDP was defined as systolic/diastolic blood pressure ≥ 140/90 mmHg after 20 weeks of gestation. Data were abstracted using REDCap and analyzed with StatCrunch. Univariable and multivariable logistic regression identified factors associated with HDP. Bivariate analyses examined associations with birth outcomes. Statistical significance was set at *p* < 0.05.

**Results:**

The prevalence of HDP was 16.8% (68/405). In multivariable analysis, increasing age (AOR = 1.31 per year, 95% CI: 1.06–1.63, *p* = 0.012), a previous history of hypertension (AOR = 1.77, 95% CI: 1.19–2.62, *p* = 0.005), and a positive family history of chronic hypertension (AOR = 3.89, 95% CI: 1.03–14.61, *p* = 0.044) were independent predictors. HDP was significantly associated with adverse birth outcomes (*p* = 0.014), particularly fetal death (46.2% vs. 16.1% among normotensive). Hypertensive adolescents had higher rates of preeclampsia (86.4% vs. 12.8%, *p* < 0.001), eclampsia (100% vs. 16%, *p* < 0.001), and fetal complications, including asphyxia (34.5% vs. 14.6%, *p* = 0.013) and prematurity (23.5% vs. 14.6%).

**Conclusions:**

HDP affect nearly one in six adolescent mothers at a Zambian tertiary hospital and are strongly associated with adverse birth outcomes. Older age, prior hypertension, and family history of chronic hypertension are independent predictors. Enhanced antenatal risk assessment, including detailed personal and family history, age‐stratified monitoring, and routine proteinuria screening, is urgently needed to improve maternal and neonatal outcomes in this high‐risk population.

## 1. Introduction

Hypertensive disorders of pregnancy (HDP) represent a leading cause of maternal and perinatal morbidity and mortality worldwide, accounting for approximately 10%–15% of all pregnancy‐related deaths [1, 2]. This clinical spectrum includes gestational hypertension (HTN), preeclampsia, eclampsia, and chronic HTN with superimposed preeclampsia [3]. Gestational HTN, defined as new‐onset systolic blood pressure ≥ 140 mmHg and/or diastolic blood pressure ≥ 90 mmHg after 20 weeks of gestation in a previously normotensive woman, is the most common form [4]. When left unrecognized or inadequately managed, it can progress to preeclampsia characterized by proteinuria and end‐organ dysfunction or to eclampsia, a life‐threatening convulsive state [5–7]. Collectively, HDP contribute substantially to adverse pregnancy outcomes, including preterm birth, low birth weight, intrauterine growth restriction, placental abruption, and stillbirth [8, 9].

Adolescent mothers, defined by the World Health Organization as girls aged 10–19 years, face a disproportionately high risk of HDP and its complications [10]. Approximately 11% of all births globally occur among adolescents, with the vast majority taking place in low‐ and middle‐income countries [11]. In Sub‐Saharan Africa, adolescent pregnancy rates remain persistently high, driven by early marriage, limited access to contraception, and socioeconomic vulnerabilities [12]. The combination of young maternal age and the physiological demands of pregnancy creates a high‐risk environment: the reproductive and cardiovascular systems of younger adolescents may not be fully mature, and the increased metabolic load can overwhelm their still‐developing vasculature [13]. Moreover, adolescent mothers often face barriers to timely antenatal care, including stigma, lack of transport, financial constraints, and limited health literacy, which delay the diagnosis and management of HDP [14].

The birth outcomes associated with HDP among adolescents are particularly concerning. Hypertensive disorders compromise placental blood flow, leading to fetal hypoxia, restricted nutrient delivery, and an increased likelihood of preterm delivery, low birth weight, and neonatal asphyxia [15, 16]. In severe cases, eclampsia and placental abruption can result in stillbirth or early neonatal death [17]. Beyond the immediate perinatal period, infants born to mothers with HDP are at greater risk of long‐term developmental and cardiovascular sequelae [8]. For the adolescent mother, a history of HDP is a strong predictor of future chronic HTN, cardiovascular disease, and recurrent hypertensive disorders in subsequent pregnancies [18–20].

Despite the high burden of adolescent pregnancy in Zambia and other low‐resource settings, there is a paucity of data specifically examining the prevalence of HDP and their associated birth outcomes among this vulnerable population. Most existing studies have either focused on adult women or have aggregated all age groups, thereby masking the unique risks faced by adolescents [21]. Furthermore, few studies have been conducted at tertiary hospitals in Zambia, where referral of complicated cases may provide a more accurate picture of severe HDP and their sequelae. Understanding the local epidemiology of HDP among adolescents is essential for designing targeted antenatal interventions, allocating resources, and training healthcare workers to recognize and manage these conditions early.

Hence, the goal of this study is to determine the prevalence of HDP among adolescent mothers (aged 10–19 years) at Livingstone University Teaching Hospital (LUTH), a high‐volume tertiary referral center in Southern Province, Zambia, and to examine the association between these disorders and adverse birth outcomes, including preterm birth, fetal distress, and perinatal mortality. By identifying the burden and consequences of HDP in this high‐risk population, the study aims to provide evidence to inform targeted antenatal interventions, strengthen adolescent‐friendly care, and guide resource allocation in low‐resource settings.

## 2. Materials and Methods

### 2.1. Study Design

This cross‐sectional study utilized hospital records to evaluate serious maternal complications associated with adolescent pregnancy (ages 10 and 19 years) at LUTH, Zambia. The study focused exclusively on maternal outcomes, excluding neonatal complications. The Strengthening the Reporting of Observational Studies in Epidemiology (STROBE) guidelines were followed to ensure methodological rigor.

### 2.2. Study Setting

The study was conducted at LUTH, a tertiary referral hospital in Southern Province, Zambia, serving a high‐risk, high‐volume obstetric population. The facility’s extensive catchment area and annual delivery load provided a robust sample for assessing adolescent pregnancy complications.

### 2.3. Eligibility and Recruitment

Out of 1200 available records, 820 files met the inclusion criteria and were screened for adolescent pregnancies (aged 10–19 years) with deliveries or admissions between January 2023 and December 2024. Of these, 405 records were eligible and included in the final analysis. A total of 413 records were excluded due to missing key information (e.g., age, sex, residence, and relevant maternal details) or incomplete clinical data, including absence of documented complications such as preterm labor, preeclampsia, and postpartum hemorrhage.

### 2.4. Data Collection

A structured retrospective review was conducted from January 2 to April 16, 2025, by trained research assistants using a standardized protocol. Data abstraction was performed via the Research Electronic Data Capture (REDCap) platform to ensure consistency and minimize entry errors.

### 2.5. Study Variables

The primary outcome variable was HTN in pregnancy, which was defined as systolic blood pressure ≥ 140 mmHg and/or diastolic blood pressure ≥ 90 mmHg on two separate readings taken at least four hours apart after 20 weeks of gestation in a previously normotensive woman [22]. Independent variables included sociodemographic: age, residence (urban/rural), employment status, and education level. Clinical: parity, gravidity, delivery mode, blood pressure, proteinuria, serum creatinine, ALT, hematological indices (WBC, RBC, hemoglobin, and platelets), and serum urea.

### 2.6. Data Analysis

Following data abstraction into REDCap, the data were exported to Microsoft Excel 2013 for cleaning and coding and subsequently analyzed using StatCrunch (https://www.statcrunch.com/). Categorical variables were summarized using frequencies and percentages, while continuous variables were described as medians with interquartile ranges (IQRs). The Shapiro–Wilk test was used to assess the normality of the data. Differences in medians between groups were evaluated using the Wilcoxon rank‐sum test, while associations between categorical variables were assessed using chi‐squared tests. Factors associated with HTN in pregnancy were examined using univariable and multivariable logistic regression analyses. Candidate variables were selected based on prior literature and clinical relevance. Variables with a univariable *p* value < 0.05 or those considered biologically important were included in the multivariable model. Statistical significance was set at *p* < 0.05.

## 3. Results

**FIGURE 1:**
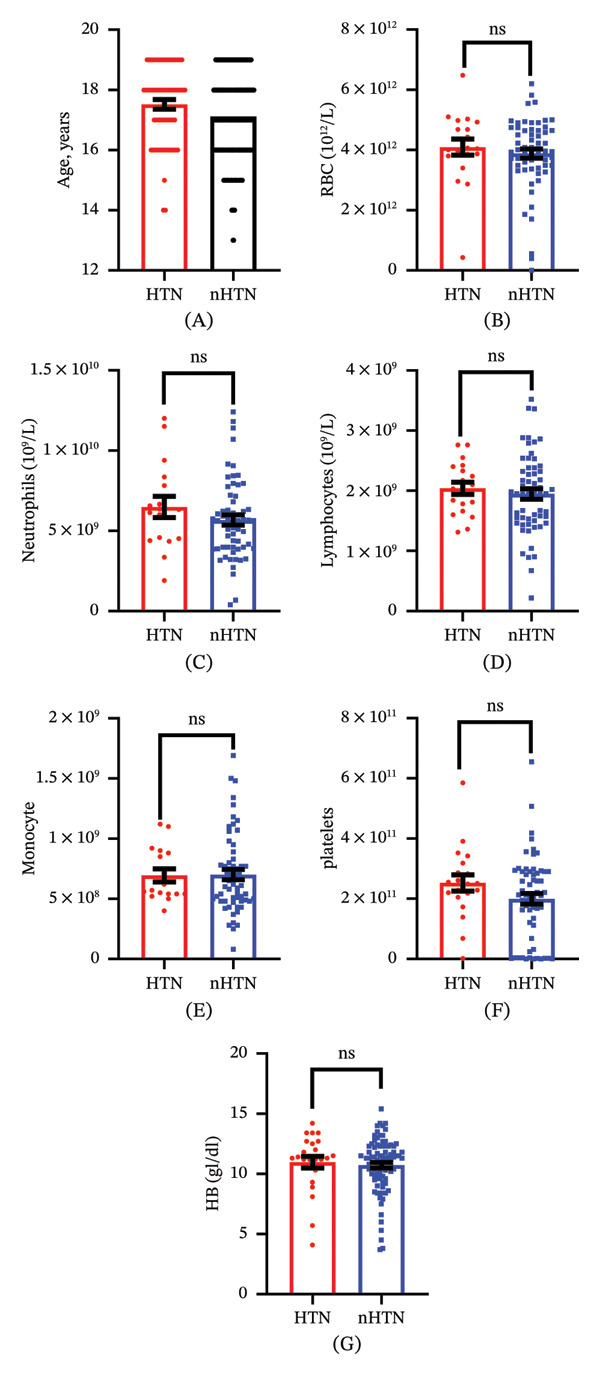
Age and complete blood count parameters of adolescents with and without gestational hypertension. (A) Age between those with gHTN (*n* = 67) and those without gHTN (*n* = 337). (B) RBCs between those with gHTN (*n* = 20) and those without gHTN (*n* = 61). (C) Neutrophils between those with gHTN (*n* = 17) and those without gHTN (*n* = 60). (D) Lymphocytes between those with gHTN (*n* = 19) and those without gHTN (*n* = 58). (E) Monocytes between those with gHTN (*n* = 17) and those without gHTN (*n* = 61). (F) Platelets between those with gHTN (*n* = 20) and those without gHTN (*n* = 62). (G) HB between those with gHTN (*n* = 24) and those without gHTN (*n* = 88). ^∗^ *p* < 0.05; ^∗∗^ *p* < 0.01; ^∗∗∗^ *p* < 0.001; ^∗∗∗∗^ *p* < 0.0001; ns, *p* > 0.05.

The study included participants with a median age of 17 years (IQR: 16–18), with hypertensive individuals being slightly older (18 vs. 17 years, *p* = 0.020) (Table 1 and Figure 1). Most participants were unmarried (83.8%), and marital status did not significantly affect HTN prevalence (*p* = 0.975). Education level showed a borderline association, with higher HTN rates among those with secondary education (22.2%) compared to primary education (13.2%, *p* = 0.054). Adverse birth outcomes, particularly fatal cases (46.2%), were strongly linked to HTN (*p* = 0.014). Current preeclampsia (86.4% vs. 12.8%, *p* < 0.0001) and eclampsia (100% vs. 16%, *p* < 0.0001) were highly associated with HTN, as was a family history of chronic HTN (40.0% vs. 16.2%, *p* = 0.046). Fetal complications, such as asphyxia (34.5%) and prematurity (23.5%), also correlated with higher HTN rates (*p* = 0.013). Proteinuria demonstrated a strong association (64.3% vs. 15.1%, *p* < 0.0001), while HIV status, smoking, and most hematological parameters showed no significant differences. However, a history of alcohol use approached significance (50.0% vs. 16.3%, *p* = 0.062). These findings highlight key risk factors, particularly pregnancy‐related complications and proteinuria, in the development of HTN in this population among adolescents.

**TABLE 1:** Relationship between demographic and clinical characteristics with hypertension.

Variable	Median (IQR) or frequency (%)	Hypertension		
		Yes = 68 (16.8%)	No = 337 (83.2)	*p* value
Marital status				0.975
Married	66 (16.2)	11 (16.9)	54 (83.1)	
Unmarried	341 (83.8)	57 (16.8)	283 (83.2)	
Residence				0.839
Rural	192 (47.7)	33 (17.2)	159 (82.8)	
Urban	213 (52.3)	35 (16.4)	178 (83.6)	
Level of education				0.054
Primary	242 (59.5)	32 (13.2)	210 (86.8)	
Secondary	164 (40.3)	36 (22.2)	126 (77.8)	
Birth outcomes				**0.014**
Term	344 (84.5)	55 (16.1)	287 (83.9)	
Preterm	50 (12.3)	7 (14.0)	43 (86.0)	
Death	13 (3.2)	7 (46.2)	7 (53.8)	
History of preeclampsia				0.652
Yes	1 (0.2)	0 (0)	1 (100)	
No	406 (99.8)	68 (16.8)	336 (83.2)	
Currently has preeclampsia				**< 0.0001**
Yes	22 (5.4)	19 (86.4)	3 (13.6)	
No	385 (94.6)	49 (12.8)	334 (87.2)	
Fetal complications				**0.0126**
None	344 (84.5)	50 (14.6)	292 (85.4)	
Prematurity	34 (8.35)	8 (23.5)	26 (76.5)	
Asphyxia	29 (7.1)	10 (34.5)	19 (65.5)	
Route of delivery				0.339
No delivery	27 (6.6)	3 (11.1)	24 (88.9)	
C/D	52 (12.8)	12 (23.1)	40 (76.90)	
NVD	328 (80.6)	53 (16.3)	273 (83.7)	
Gravidity	1 (1.1)	1 (1.1)	1 (1.1)	0.169
Parity	1 (1.1)	1 (1.1)	1 (1.1)	0.750
Eclampsia				**< 0.0001**
Yes	4 (1.2)	4 (100.0)	0 (0.0)	
No	403 (98.8)	64 (16.0)	337 (84.0)	
Family history of chronic hypertension				**0.046**
Yes	10 (2.5)	4 (16.2)	6 (60.0)	
No	397 (97.5)	64 (16.2)	331 (84.0)	
Living with HIV				1.000
Yes	9 (2.2)	1 (12.5)	7 (87.5)	
No	398 (97.8)	67 (16.9)	330 (83.1)	
History of smoking				0.308
Yes	2 (0.49)	1 (50.0)	1 (50.0)	
No	405 (99.5)	67 (16.6)	336 (83.4)	
Currently smoking				0.308
Yes	2 (0.49)	1 (50.0)	1 (50.0)	
No	405 (99.5)	67 (16.6)	336 (83.4)	
History of alcohol				0.062
Yes	6 (1.47)	3 (50.0)	3 (50.0)	
No	401 (98.5)	65 (16.3)	334 (83.7)	
Proteinuria on dipstick				**< 0.0001**
Yes	14 (3.4)	9 (64.3)	5 (35.7)	
No	393 (96.6)	59 (15.1)	332 (84.9)	

*Note:* Bold values mean the p value is statistically significant or ^∗^ns (*p* > 0.05); ^∗^ *p* < 0.05; ^∗∗^ *p* < 0.01; ^∗∗∗^ *p* < 0.001; ^∗∗∗^ *p* < 0.0001.

Abbreviations: C/V, cesarean delivery; g/dL, grams per deciliter; IQR, interquartile range; NVD, normal vaginal delivery; RBC, red blood cell.

### 3.1. Interpretation of Results in Figure 1

The results show some differences between participants with HTN and those without (normotensive). Individuals in the hypertensive group were slightly older than those in the normotensive group, and this difference was statistically significant (^∗^ *p* < 0.05). This suggests that age may play a role in the occurrence of HTN in this population. However, for the other blood‐related parameters, there were no meaningful differences between the two groups. Red blood cell counts, neutrophils, lymphocytes, monocytes, platelets, and hemoglobin levels were generally similar among participants with and without HTN (all *p* > 0.05). Although there was some variation within each group, the overall patterns were comparable.

### 3.2. Regression Analysis of Factors Associated With Hypertension in Adolescent Mothers

In the univariable analysis, increasing age was associated with higher odds of HTN, with each additional year linked to a 26% increase in the odds (OR = 1.26; 95% CI: 1.03–1.54; *p* = 0.022) (Table 2). A previous history of HTN also significantly increased the odds of developing HTN, with affected adolescents showing a 75% higher likelihood compared to those without prior HTN (OR = 1.75; 95% CI: 1.19–2.59; *p* = 0.004). Family history of HTN showed a positive but borderline association (OR = 3.44; 95% CI: 0.94–12.56; *p* = 0.060), while parity, gravidity, and marital status were not significantly associated with HTN in the univariable analysis.

**TABLE 2:** Logistic regression analysis of demographic and clinical characteristics associated with hypertension in adolescent pregnancy.

Variable	Univariable analysis		Multivariable analysis	
	OR (95% CI)	*p* value	OR (95% CI)	*p* value
Age	1.26 (1.03, 1.54)	**0.022**	1.31 (1.06, 1.63)	**0.012**
Family history of HTN				
No	REF	REF	REF	REF
Yes	3.44 (0.94, 12.56)	0.060	3.89 (1.03, 14.61)	**0.044**
Parity	1.06 (0.51, 2.21)	0.868	1.09 (0.52, 2.28)	0.800
Gravidity	0.28 (0.03, 2.14)	0.220	0.22 (0.02, 1.72)	0.151
Previous HTN				
No	REF	REF	REF	REF
Yes	1.75 (1.19, 2.59)	**0.004**	1.77 (1.19, 2.62)	**0.005**
Marital status				
Unmarried	REF	REF	REF	REF
Married	1.01 (0.49, 2.05)	0.975	0.72 (0.32, 1.62)	0.435

*Note:* REF, reference. Bold values mean the *p* value is statstically significant or ^∗^ns (*p* > 0.05); ^∗^ *p* < 0.05; ^∗∗^ *p* < 0.01; ^∗∗∗^ *p* < 0.001; ^∗∗∗^ *p* < 0.0001.

Abbreviations: AOR, adjusted odds ratio; OR, odds ratio.

In the multivariable model, age remained an independent predictor, with each additional year of age increasing the odds of HTN by 31% (AOR = 1.31; 95% CI: 1.06–1.63; *p* = 0.012). A previous history of HTN continued to be a significant predictor (AOR = 1.77; 95% CI: 1.19–2.62; *p* = 0.005). In addition, family history of HTN emerged as an independent risk factor after adjustment for confounders, with adolescents reporting a positive family history having nearly four times the odds of HTN compared to those without such a history (AOR = 3.89; 95% CI: 1.03–14.61; *p* = 0.044). Parity, gravidity, and marital status were not significant in the multivariable model, indicating that age, personal history of HTN, and familial predisposition were the primary independent predictors of hypertensive disorders among adolescent mothers.

## 4. Discussion

Our study aimed at establishing the prevalence of HTN in adolescent’s mothers visiting LUTH. We found the prevalence of HTN in adolescent mothers to be 16.8%, with a statistically significant difference between groups (*p* value = 0.0202). While the majority of existing studies have not explored HTN prevalence in adolescents, our research addresses this gap**.** The study finding falls within the lower range of reported HTN prevalence in various studies. For example, systematic reviews suggest that HTN among healthcare workers ranges from 13% to 40%, depending on factors such as age, BMI, and occupational stress [23]. Similarly, a meta‐analysis found an overall HTN prevalence of 30.5% in urban areas and 27.9% in rural areas in low‐ and middle‐income countries, showing a slight urban–rural disparity [24]. A global projection study estimated a decline in HTN prevalence from 22.1% in 2015 to 20.3% by 2040, with low‐income countries expected to have the highest rates [23–25]. Although these findings may suggest that the study population had fewer risk factors or benefited from better health management compared to broader populations, the observed statistically significant differences highlight the need for further investigation into underlying contributors, including socioeconomic status, lifestyle factors, and access to healthcare.

Our study also revealed that as age increases within the adolescent group, the risk of developing HTN during pregnancy also rises. This aligns with a study by Datta et al., which indicated that an increase in age in pregnancy was associated with HTN [26]. In addition, another study by Ratnam et al. in Malaysia revealed that as age increased in adolescents, the likelihood of having HTN increased [27]. These findings may reflect cumulative biological and social exposures, such as higher parity or increased stress, which are more common in older adolescents [28]. Similar patterns have been documented in other settings. For example, research from South Africa found that older adolescents were more prone to pregnancy‐induced HTN than younger ones, suggesting a gradient of risk within adolescence itself [29]. A study from India also supports this association, highlighting the influence of age even within the adolescent group and emphasizing that the timing and pattern of adolescent childbearing may play an important role in shaping hypertensive risk [26]. Furthermore, the progressive increase in the risk of HTN with age among adolescents suggests that late adolescence (18–19 years) carries a distinct vulnerability compared to early adolescence. This pattern has been observed in other settings, with older adolescent mothers being more likely to develop hypertensive disorders during pregnancy, independent of factors such as parity or socioeconomic status [30, 31]. The relationship may reflect cumulative psychosocial stress, longer exposure to high‐risk behaviors, and subtle biological changes that occur as adolescents mature [32, 33]. In addition, from a physiological perspective, younger adolescents may exhibit greater vascular compliance and lower baseline sympathetic activity, which could confer relative protection against the hemodynamic stresses of pregnancy. Conversely, older adolescents may demonstrate early manifestations of endothelial dysfunction, particularly in the context of multiparity or suboptimal nutritional status [34, 35].

A prior history of HTN, whether chronic essential HTN or a previous hypertensive disorder of pregnancy, represents one of the most potent predictors of recurrent hypertensive complications [36]. Supporting this, a meta‐analysis by Brouwers et al. reported that women with a history of preeclampsia or gestational HTN in a previous pregnancy faced an approximately sevenfold increased risk of recurrence [37]. Among adolescents, Parra‐Pingel and colleagues reported that those with a prior history of severe preeclampsia had significantly higher rates of adverse maternal outcomes in subsequent pregnancies, even when the index pregnancy occurred in adolescence [38]. Furthermore, adolescent mothers with a history of HTN in a previous pregnancy, particularly when pregnancies occur within a short interpregnancy interval, may face a considerable cumulative burden on both cardiovascular and renal function [39]. This suggests that young women should be managed as high risk from the outset, with early initiation of blood pressure monitoring.

Finally, a history of chronic HTN emerged as another significant predictor. The shift in statistical significance indicates potential negative confounding; adolescents with a positive family history may have also possessed protective characteristics, such as younger age or lower parity, which initially obscured the true effect. After adjusting for these confounders, the independent contribution of genetic predisposition to hypertensive risk became apparent. Our findings align with several studies that have identified family history as an important risk factor for HDP, even among adolescents. A study by Bune in Ethiopia similarly reported that having a first‐degree relative with HTN was associated with an increased risk of developing pregnancy‐induced HTN among adolescent mothers [40]. Similarly, a systematic review by Macedo et al. noted that family history of HTN or preeclampsia consistently emerged as a risk factor for preeclampsia across diverse populations, including adolescent cohorts [24]. The biological basis likely involves shared genetic variants affecting the renin–angiotensin–aldosterone system (RAAS), endothelial function, and inflammatory pathways [41, 42]. The emergence of family history as a significant factor only after adjustment highlights an important methodological consideration. Relying solely on univariable analyses may lead to the exclusion of important predictors that are masked by confounding. From a clinical perspective, this finding emphasizes the importance of obtaining a comprehensive family history during the initial antenatal visit. Adolescents with a first‐degree relative, such as a mother or sister, who has a history of HTN, should be considered at increased risk, even in the absence of prior complications or younger maternal age.

### 4.1. Strengths and Limitations

This study has several notable strengths. It addresses an important evidence gap by focusing on HTN among adolescent mothers in a low‐resource, high‐burden setting in Sub‐Saharan Africa. The relatively large sample size and use of data from a high‐volume tertiary referral hospital enhanced the study’s statistical power and ability to detect meaningful associations. Data collection through a standardized REDCap protocol improved data quality and consistency, while the inclusion of both sociodemographic and clinical variables allowed for a comprehensive analysis. In addition, the use of multivariable logistic regression enabled the identification of independent predictors while controlling for confounding, and adherence to STROBE guidelines supports methodological rigor and transparency. However, several limitations should be considered. The retrospective cross‐sectional design limits causal inference, and the single‐center setting may reduce generalizability, as the study population likely represents higher‐risk cases. Reliance on medical records introduces the potential for incomplete or inaccurate data, particularly for variables such as family history and behavioral factors. The wide confidence interval observed for family history of HTN suggests limited precision due to small subgroup sizes. Furthermore, the study did not stratify adolescents into early and late age groups or account for important confounders such as body mass index, nutritional status, interpregnancy interval, and antenatal care utilization. The inability to clearly distinguish between chronic and gestational HTN, along with the lack of detailed neonatal outcomes, further limits interpretation. Despite these limitations, the study provides valuable insight into the burden and determinants of gestational HTN among adolescent mothers in Zambia and highlights key areas for future research and targeted interventions.

## 5. Conclusion

This study reported a 16.8% prevalence of HTN among adolescent mothers at LUTH, Zambia, indicating that approximately one in six adolescent pregnancies is affected. Increasing age, a prior history of HTN, and a positive family history of chronic HTN were identified as independent predictors. However, the wide confidence interval observed for family history suggests limited precision and highlights the need for confirmation in larger studies. These findings emphasize the importance of strengthened antenatal risk assessment, including detailed personal and family history, age‐specific monitoring, and routine screening for proteinuria. Targeted interventions for older adolescents and those with prior or familial risk factors are critical to reducing maternal and neonatal morbidity in low‐resource settings. Future prospective multicenter studies are warranted to validate these findings and inform effective prevention strategies.

## Author Contributions

M.C. and S.K.M. conceived the study. S.K.M. and M.C. oversaw data acquisition. S.K.M. and M.C. supervised data acquisition. S.K.M. and M.C. conducted the formal analysis. M.C., D.C., H.H., L.S., J.P.P., N.C., A.K., and S.K.M. wrote the original draft.

## Funding

No funding was received.

## Disclosure

All authors contributed to the article edits and approved the final manuscript.

## Ethics Statement

Ethical approval was granted by the Mulungushi University School of Medicine and Health Sciences Research Ethics Committee (Ref: SMHS‐MU2‐2024‐238) on May 10, 2024. In accordance with the Declaration of Helsinki on human research ethics, records from all the consenting participants were de‐identified to protect confidentiality, and no personally identifiable information was collected. As the study utilized retrospective, anonymized records, informed consent was waived by the ethics committee.

## Conflicts of Interest

The authors declare no conflicts of interest.

## Data Availability

The raw data supporting this study’s findings are available from the corresponding author upon reasonable request, without undue restriction.

## References

[1] Khedagi A. M. and Bello N. A. , Hypertensive Disorders of Pregnancy, Cardiology Clinics. (2021) 39, no. 1, 77–90, 10.1016/j.ccl.2020.09.005. 33222817 PMC7720658

[2] Khan Z. L. , Balie G. M. , and Chauke L. , Hypertensive Disorders of Pregnancy Deaths: A Four-Year Review at a Tertiary/Quaternary Academic Hospital, International Journal of Environmental Research and Public Health. (2025) 22, no. 7, 10.3390/ijerph22070978. PMC12294271 40724045

[3] Braunthal S. and Brateanu A. , Hypertension in Pregnancy: Pathophysiology and Treatment, SAGE Open Medicine. (2019) 7, 10.1177/2050312119843700. PMC6458675 31007914

[4] Porcelli B. A. , Diveley E. , Meyenburg K. et al., A New Definition of Gestational Hypertension? New-Onset Blood Pressures of 130 to 139/80 to 89 Mm Hg After 20 Weeks of Gestation, American Journal of Obstetrics and Gynecology. (2020) 223, no. 3, 442.e1–442.e7, 10.1016/j.ajog.2020.06.019. 32553915

[5] WHO , Pre-Eclampsia, 2025, https://www.who.int/news-room/fact-sheets/detail/pre-eclampsia.

[6] Chang K.-J. , Seow K.-M. , and Chen K.-H. , Preeclampsia: Recent Advances in Predicting, Preventing, and Managing the Maternal and Fetal Life-Threatening Condition, International Journal of Environmental Research and Public Health. (2023) 20, no. 4, 10.3390/ijerph20042994. PMC9962022 36833689

[7] Chakulya M. , Mulambo P. , Chama G. C. et al., Preeclampsia, Prevalence and Associated Factors, PLoS One. (2025) 20, no. 12, 10.1371/journal.pone.0337190. PMC12677571 41343536

[8] Sokou R. , Lianou A. , Lampridou M. et al., Neonates at Risk: Understanding the Impact of High-Risk Pregnancies on Neonatal Health, Medicina. (2025) 61, no. 6, 10.3390/medicina61061077. PMC12194930 40572764

[9] Qiang Y. , Xiaoli D. , and Nawsherwan L. Q. , Prevalence, Incidence, and Temporal Trend of Hypertensive Disorders of Pregnancy and Its Association with Adverse Perinatal Outcomes in High and Low-Middle Income Countries: a Narrative Review, Iranian Journal of Public Health. (2025) 54, 701–709, 10.18502/ijph.v54i4.18409. 40321929 PMC12045870

[10] WHO , Adolescent Pregnancy, 2024, https://www.who.int/news-room/fact-sheets/detail/adolescent-pregnancy.

[11] Diabelková J. , Rimárová K. , Dorko E. , Urdzík P. , Houžvičková A. , and Argalášová Ľ. , Adolescent Pregnancy Outcomes and Risk Factors, International Journal of Environmental Research and Public Health. (2023) 20, no. 5, 10.3390/ijerph20054113. PMC10002018 36901128

[12] Maharaj N. R. , Adolescent Pregnancy in Sub-Saharan Africa–A Cause for Concern, Frontiers in Reproductive Health. (2022) 4, 10.3389/frph.2022.984303. PMC9755883 36531444

[13] Malunga G. , Sangong S. , Saah F. I. , and Bain L. E. , Prevalence and Factors Associated With Adolescent Pregnancies in Zambia: A Systematic Review From 2000–2022, Archives of Public Health. (2023) 81, no. 1, 10.1186/s13690-023-01045-y. PMC9940412 36805786

[14] Nguru M. C. , Maroyi R. , Ganywamulume B. , Maha A. B. , Ngeleza O. N. , and Mukwege D. M. , Challenges and Prospects in Managing Hypertensive Disorders of Pregnancy in Sub-Saharan Africa: A Narrative Review, International Journal of Women’s Health. (2026) 18, 10.2147/IJWH.S593155. PMC12985167 41835850

[15] Zerihun E. , Girma F. , Amena N. et al., Effect of Hypertensive Disorders of Pregnancy (HDP) on Maternal and Perinatal Birth Outcomes in Eastern Ethiopia: a Prospective Cohort Study, BMC Pregnancy and Childbirth. (2025) 25, no. 1, 10.1186/s12884-025-07707-9. PMC12105356 40420004

[16] Li Q. , Wu T. , Mao Y. et al., Complications in Hospitalized Neonates Born to Mothers with HDP Subtypes: A Retrospective Cohort Study and Mediation Analysis, BMC Pregnancy and Childbirth. (2025) 25, no. 1, 10.1186/s12884-025-07778-8. PMC12145605 40483435

[17] Basta M. , Hanif K. , Zafar S. et al., Impact of Hypertensive Disorders of Pregnancy on Stillbirth and Other Perinatal Outcomes: A Multi-Center Retrospective Study, Cureus. (2022) 14, 10.7759/cureus.22788. PMC8986463 35399480

[18] Jaleta D. D. , Gebremedhin T. , and Jebena M. G. , Perinatal Outcomes of Women With Hypertensive Disorders of Pregnancy in Jimma Medical Center, Southwest Ethiopia: Retrospective Cohort Study, PLoS One. (2021) 16, no. 8, 10.1371/journal.pone.0256520. PMC8375998 34411170

[19] von Dadelszen P. and Magee L. A. , Preventing Deaths due to the Hypertensive Disorders of Pregnancy, Best Practice & Research Clinical Obstetrics & Gynaecology. (2016) 36, 83–102, 10.1016/j.bpobgyn.2016.05.005. 27531686 PMC5096310

[20] Melchiorre K. , Thilaganathan B. , Giorgione V. , Ridder A. , Memmo A. , and Khalil A. , Hypertensive Disorders of Pregnancy and Future Cardiovascular Health, Frontiers in Cardiovascular Medicine. (2020) 7, 10.3389/fcvm.2020.00059. PMC7174679 32351977

[21] Gemechu K. S. , Assefa N. , and Mengistie B. , Prevalence of Hypertensive Disorders of Pregnancy and Pregnancy Outcomes in Sub-Saharan Africa: A Systematic Review and meta-analysis, Women’s Health. (2020) 16, 10.1177/1745506520973105. PMC7750906 33334273

[22] WHO. Hypertension, 2023, https://www.who.int/news-room/fact-sheets/detail/hypertension.

[23] Boateng E. B. and Ampofo A. G. , A Glimpse Into the Future: Modelling Global Prevalence of Hypertension, BMC Public Health. (2023) 23, no. 1, 10.1186/s12889-023-16662-z. PMC10546636 37789258

[24] Macedo T. C. C. , Montagna E. , Trevisan C. M. et al., Prevalence of Preeclampsia and Eclampsia in Adolescent Pregnancy: A Systematic Review and Meta-Analysis of 291,247 Adolescents Worldwide Since 1969, European Journal of Obstetrics & Gynecology and Reproductive Biology. (2020) 248, 177–186, 10.1016/j.ejogrb.2020.03.043. 32283429

[25] Kraft P. and Kraft B. , Explaining Socioeconomic Disparities in Health Behaviours: A Review of Biopsychological Pathways Involving Stress and Inflammation, Neuroscience & Biobehavioral Reviews. (2021) 127, 689–708, 10.1016/j.neubiorev.2021.05.019. 34048858

[26] Datta B. K. , Husain M. J. , and Kostova D. , Hypertension in Women: The Role of Adolescent Childbearing, BMC Public Health. (2021) 21, no. 1, 10.1186/s12889-021-11488-z. PMC8323295 34325686

[27] Ratnam K. K. Y. , Suliman M. A. B. , Sui W. K. , Tok P. S. K. , and Yusoff M. F. B. M. , Prevalence of Hypertension in Pregnancy and Its Associated Sociodemographic Factors Among Mothers Aged 15–49 Years Old in Malaysia, Archives of Public Health. (2024) 82, no. 1, 10.1186/s13690-024-01349-7. PMC11318284 39135192

[28] Eiden R. D. , Ettekal I. , Zhao J. et al., Prenatal Substance Exposure, Early Life Adversity, and Parenting: Associations with Adolescent Stress Response, Developmental Psychobiology. (2023) 65, no. 2, 10.1002/dev.22365. PMC9971663 36811371

[29] Peter B. B. and Okafor U. B. , Pregnancy-Induced Hypertension Awareness, Knowledge and Its Risk Factors: a cross-sectional Study, Pakistan Journal of Medical Sciences. (2024) 40, no. 4, 629–636, 10.12669/pjms.40.4.8247. 38544992 PMC10963995

[30] Ordoñez-Villordo E. , Cureño-Díaz M. A. , Gómez-Zamora E. et al., Age at Menarche and Risk of Hypertensive Disorders of Pregnancy: A Retrospective Cohort Study, Clinical Practice. (2026) 16, no. 2, 10.3390/clinpract16020032. PMC12939861 41744516

[31] Nath A. , Sheeba B. , Sisira R. , and Metgud C. S. , Prevalence of Hypertension in Pregnancy and Its Associated Factors Among Women Attending Antenatal Clinics in Bengaluru, Journal of Family Medicine and Primary Care. (2021) 10, no. 4, 1621–1627, 10.4103/jfmpc.jfmpc_1520_20. PMC8144779 34123902

[32] Parise L. F. , Joseph Burnett C. , and Russo S. J. , Early Life Stress and Altered Social Behaviors: A Perspective Across Species, Neuroscience Research. (2025) 211, 65–74, 10.1016/j.neures.2023.11.005. 37992997 PMC11102940

[33] Schmitt S. A. , Paes T. M. , Duncan R. J. , and Vandell D. L. , Early Cumulative Risk and Outcomes in Adolescence and Adulthood: The Role of Executive Function and Behavioral Regulation, Developmental Psychology. (2023) 59, no. 11, 1988–2001, 10.1037/dev0001647. 37768603 PMC10841203

[34] Spradley F. T. , Sympathetic Nervous System Control of Vascular Function and Blood Pressure During Pregnancy and Preeclampsia, Journal of Hypertension. (2019) 37, no. 3, 476–487, 10.1097/HJH.0000000000001901. 30160658 PMC6355368

[35] Macro L. E. , von Ah Morano A. E. , Halligan S. L. , and Barker A. R. , Associations Between Adverse Childhood Experiences and Vascular Indicators of Atherosclerosis Measured in Childhood and Early to Mid-Adulthood: A Systematic Review, Social Science & Medicine. (2025) 384, 10.1016/j.socscimed.2025.118515. 40886525

[36] Traub A. , Sharma A. , and Gongora M. C. , Hypertensive Disorders of Pregnancy: A Literature Review–Pathophysiology, Current Management, Future Perspectives, and Healthcare Disparities, US Cardiology Review. (2024) 18, 10.15420/usc.2023.01. PMC11526487 39494413

[37] Brouwers L. , van der Meiden-van Roest A. , Savelkoul C. et al., Recurrence of Pre-Eclampsia and the Risk of Future Hypertension and Cardiovascular Disease: A Systematic Review and Meta-Analysis, BJOG. (2018) 125, no. 13, 1642–1654, 10.1111/1471-0528.15394. 29978553 PMC6283049

[38] Parra-Pingel P. E. , Quisiguiña-Avellán L. A. , Hidalgo L. , Chedraui P. , and Pérez-López F. R. , Pregnancy Outcomes in Younger and Older Adolescent Mothers With Severe Preeclampsia, Adolescent Health, Medicine and Therapeutics. (2017) 8, 81–86, 10.2147/AHMT.S131050. 28652838 PMC5476435

[39] Turi V.-R. , Luca C. T. , Gaita D. et al., Diagnosing Arterial Stiffness in Pregnancy and Its Implications in the Cardio-Renal-Metabolic Chain, Diagnostics. (2022) 12, no. 9, 10.3390/diagnostics12092221. PMC9497660 36140621

[40] Bune G. T. , Pregnancy-Induced Hypertensive Disorders Predictors Among Pregnant and Delivery Mothers Receiving Care in Public Health Institutions in Sidama, Ethiopia: A Multicenter Case Control Study, BMC Pregnancy and Childbirth. (2024) 24, no. 1, 10.1186/s12884-024-06886-1. PMC11490123 39425089

[41] Valentini A. , Heilmann R. M. , Kühne A. , Biagini L. , De Bellis D. , and Rossi G. , The Renin–Angiotensin–Aldosterone System (RAAS): Beyond Cardiovascular Regulation, Veterinary Sciences. (2025) 12, no. 8, 10.3390/vetsci12080777. PMC12390185 40872727

[42] Li Y. , Yan Z. , Chaudhry K. , and Kazlauskas A. , The Renin-Angiotensin-Aldosterone System (RAAS) Is One of the Effectors by Which Vascular Endothelial Growth Factor (VEGF)/Anti-VEGF Controls the Endothelial Cell Barrier, American Journal of Pathology. (2020) 190, no. 9, 1971–1981, 10.1016/j.ajpath.2020.06.004. 32590003 PMC7538812

